# Glutamatergic Receptors Modulate Normoxic but Not Hypoxic Ventilation and Metabolism in Naked Mole Rats

**DOI:** 10.3389/fphys.2019.00106

**Published:** 2019-02-18

**Authors:** Yvonne A. Dzal, Allison Seow, Lisa G. Borecky, Danielle Chung, Sharn K. G. Gill, William K. Milsom, Matthew E. Pamenter

**Affiliations:** ^1^Department of Zoology, University of British Columbia, Vancouver, BC, Canada; ^2^Department of Biology, Centre for Forest Interdisciplinary Research, University of Winnipeg, Winnipeg, MB, Canada; ^3^Department of Biology, University of Ottawa, Ottawa, ON, Canada; ^4^University of Ottawa Brain and Mind Research Institute, Ottawa, ON, Canada

**Keywords:** ventilatory acclimatization to hypoxia, AMPA receptor, NMDA receptor, hypoxic ventilatory response, hypoxic metabolic response

## Abstract

Naked mole rats (*Heterocephalus glaber*) are among the most hypoxia-tolerant mammals, but their physiological responses to acute and chronic sustained hypoxia (CSH), and the molecular underpinnings of these responses, are poorly understood. In the present study we evaluated the acute hypoxic ventilatory response and the occurrence of ventilatory acclimatization to hypoxia following CSH exposure (8–10 days in 8% O_2_) of naked mole rats. We also investigated the role of excitatory glutamatergic signaling in the control of ventilation and metabolism in these conditions. Animals acclimated to normoxia (control) or CSH and then exposed to acute hypoxia (7% O_2_ for 1 h) exhibited elevated tidal volume (V_T_), but decreased breathing frequency (f_R_). As a result, total ventilation ($\dot{\text{V}}$_E_) remained unchanged. Conversely, V_T_ was lower in CSH animals relative to controls, suggesting that there is ventilatory plasticity following acclimatization to chronic hypoxia. Both control and CSH-acclimated naked mole rats exhibited similar 60–65% decreases in O_2_ consumption rate during acute hypoxia, and as a result their air convection requirement (ACR) increased ∼2.4 to 3-fold. Glutamatergic receptor inhibition decreased f_R_, $\dot{\text{V}}$_E_, and the rate of O_2_ consumption in normoxia but did not alter these ventilatory or metabolic responses to acute hypoxia in either the control or CSH groups. Taken together, these findings indicate that ventilatory acclimatization to hypoxia is atypical in naked mole rats, and glutamatergic signaling is not involved in their hypoxic ventilatory or metabolic responses to acute or chronic hypoxia.

## Introduction

For most adult mammals, the hypoxic ventilatory response consists of a reflex increase in ventilation ($\dot{\text{V}}$_E_) that occurs in response to the detection of decreased arterial O_2_ tension (Pa_O2_) by peripheral chemoreceptors (Powell et al., 1998; Dzal et al., 2015; Pamenter and Powell, 2016). Furthermore, with chronic sustained hypoxia (CSH) of days to months, additional time-dependent increases in $\dot{\text{V}}$_E_ occur that further improve Pa_O2_. This secondary increase is termed ventilatory acclimatization to hypoxia and persists transiently after the removal of hypoxic stimulation, indicating plasticity within the ventilatory control circuits (Aaron and Powell, 1993; Hupperets et al., 2004). In adult mammals, two mechanisms have been identified that contribute to ventilatory acclimatization to hypoxia: (1) the sensitivity of carotid body arterial chemoreceptors to O_2_ increases, and (2) the central nervous system (CNS) responsiveness to afferent inputs from the carotid bodies increase (i.e., secondary gain) (Bisgard and Neubauer, 1995; Dwinell and Powell, 1999; Wilkinson et al., 2010; Kumar and Prabhakar, 2012).

In the CNS, carotid body afferent neurons project to the *nucleus tractus solitarii* (NTS) in the brainstem (Lipski et al., 1977; Housley and Sinclair, 1988). The secondary gain within the CNS is primarily mediated by increased phosphorylation of glutamatergic α-amino-3-hydroxy-5-methyl-4-isoxazolepropionic acid and N-methyl-D-aspartate receptors (AMPARs and NMDARs, respectively) within the NTS (Connelly et al., 1992; Vardhan et al., 1993; Zhang and Mifflin, 1993; Mizusawa et al., 1994; Soto-Arape et al., 1995; Ohtake et al., 1998; Haibara et al., 1999; Reid and Powell, 2005; Braga et al., 2007; El Hasnaoui-Saadani et al., 2007; Pamenter et al., 2014a,c, 2015b). Collectively, these studies strongly support a primary role for excitatory glutamatergic signaling in mediating the acute hypoxic ventilatory response, as well as ventilatory acclimatization to hypoxia following CSH in adult mammals.

Conversely, decreases of $\dot{\text{V}}$_E_ are typically observed during steady state acute hypoxic conditions in neonatal mammals (Mortola et al., 1989; Mortola, 1999). This response is primarily mediated by inhibitory adenosinergic signaling (Elnazir et al., 1996; Johansson et al., 2001). The falls in $\dot{\text{V}}$_E_ with acute hypoxic exposure in neonates are often associated with corresponding falls in O_2_ consumption rates ($\dot{\text{V}}$_O2_) of a greater magnitude (Teppema and Dahan, 2010).

Recently, we demonstrated that adult naked mole rats (*Heterocephalus glaber*) present a neotenic phenotype in their ventilatory response to both acute hypoxia and to CSH. Specifically, $\dot{\text{V}}$_E_ decreased ∼70% in adult naked mole rats in acute hypoxia (7% O_2_) and this acute decrease was mediated by an increase in inhibitory adenosinergic signaling (Pamenter et al., 2014b, 2015a). In addition, naked mole rats also decreased $\dot{\text{V}}$_O2_ by >70 in 7% O_2_ (Pamenter et al., 2014b, 2015a, 2018a), and decreased behavioral activity in acute hypoxia (Ilacqua et al., 2017; Houlahan et al., 2018; Kirby et al., 2018). Furthermore, we found that naked mole rats did not exhibit any ventilatory plasticity following 8–10 days of acclimation to CSH (8% O_2_) (Chung et al., 2016), suggesting an absence of ventilatory acclimatization to hypoxia. Given the predominant role for glutamatergic signaling in the acute hypoxic ventilatory response and ventilatory acclimatization to hypoxia in other adult mammals, this lack of plasticity in naked mole rats implies a lack of involvement of glutamatergic signaling in ventilatory responses to hypoxia in this species, which would be a unique response amongst adult mammals.

Naked mole rats are the most hypoxia-tolerant mammal presently identified and tolerate minutes of complete anoxia, hours at 3% O_2_, and days to weeks at 8% O_2_ (Pamenter et al., 2015a, 2018b; Chung et al., 2016; Park et al., 2017). Adult naked mole rats express NMDARs, but with subunit compositions that are more typical of neonatal rodents than that of adults (Peterson et al., 2012). Furthermore, naked mole rat nervous cells demonstrate a very protracted developmental period and retain a neonatal phenotype well into adulthood (Penz et al., 2015). Given this developmental phenotype, the lack of ventilatory plasticity (i.e., ventilatory acclimatization to hypoxia), and our earlier findings of a primary role for adenosine in mediating the acute decline in ventilation in response to hypoxia, we hypothesized that the ventilatory response of naked mole rats is mediated primarily by neonate-like molecular signaling pathways. Thus, we predicted that glutamatergic signaling would not play an important role in the ventilatory responses to acute hypoxia or CSH in adult naked mole rats. Specifically, we predicted that antagonists of AMPARs or NMDARs would not have an impact on the naked mole rat hypoxic ventilatory response. To test our hypothesis, we exposed naked mole rats to 8–10 days of normoxia (control) or CSH and then examined their ventilatory and metabolic responses to an acute hypoxic challenge (7% O_2_ for 1 h), before and after glutamate receptor manipulation or sham injections of saline.

## Materials and Methods

### Animals

Naked mole rats were group-housed in interconnected multi-cage systems at 30°C and 21% O_2_ in 70% humidity with a 12L:12D light cycle. Animals were fed fresh tubers, vegetables, fruit and Pronutro cereal supplement *ad libitum*. Animals were not fasted prior to experimental trials. All experimental procedures were approved by the University of Ottawa or the University of British Columbia Animal Care Committees in accordance with the Animals for Research Act and by the Canadian Council on Animal Care. All experiments were performed during daylight hours in the middle of the animals’ 12L:12D light cycle when the animals were awake and active. Naked mole rats that are housed within colony systems, as are our experimental animals, do not exhibit circadian rhythmicity of general locomotor activity (Riccio and Goldman, 2000b), and exhibit inconsistent rhythmicity of body temperature and metabolic rate (Riccio and Goldman, 2000a); significant changes in these latter parameters were only reported in animals during the nocturnal phase of their circadian cycle with no significant changes observed during the daylight period of this cycle. Therefore, we our results should not be confounded by circadian rhythms. We examined physiological responses to environmental hypoxia in non-breeding naked mole rats. Non-breeding (subordinate) naked mole rats do not undergo sexual development or express sexual hormones and thus we did not take sex into consideration when evaluating our results (Holmes et al., 2009).

### Whole-Body Plethysmography and Respirometry

Seventy-five adult, male and female naked mole rats, weighing 44.0 ± 1.5 g (mean ± SEM) were individually placed, unrestrained, inside a 450 ml Plexiglas experimental chamber (the animal chamber), which was in turn placed into an environmental chamber held at ∼29°C. The temperature of the animal chamber was recorded continuously throughout the experiment using multiple iButtons that recorded ambient temperature at a frequency of one measurement per minute (Maxim Integrate, Chandler, CA, United States). Body temperature was measured every 10 min from subcutaneous radio frequency identification (RFID) microchips using a RFID reader (Destron Fearing, Dallas, TX, United States). In normoxia and at an ambient temperature of ∼29.0°C, the naked mole rat body temperature was 32.0 ± 0.2°C (data not shown). During acute hypoxia (1 h in 7% O_2_), body temperature decreased to 30.5 ± 0.2°C, consistent with recent measurements from our laboratory (Ilacqua et al., 2017; Kirby et al., 2018). Body temperature of CSH animals in normoxia was not different from that of control animals (32.4 ± 0.4°C, data not shown). Finally, body temperature was not altered by sham or drug injections in control or CSH animals.

Animals were provided with a thin layer of bedding on the floor of the experimental chambers. The animal chamber was sealed and constantly ventilated with gas mixtures, set to the desired fractional gas composition by calibrated rotameters (Praxair, Mississauga, ON, CA, United States). The advantage of this open-flow system is that it prevents the depletion of O_2_ and accumulation of metabolic CO_2_ by flushing the animal chamber with fresh gas, and it allows for continuous and simultaneous monitoring of metabolic and ventilatory variables. Inflowing gas was provided at a flow rate of 110 ml/min, as assessed by a calibrated mass flow meter (Alicat Scientific, Tuscon, AZ, United States). The analyzers were calibrated prior to each trial with 20.95% O_2_, 1.5% CO_2_, balance N_2_, and with 100% N_2_ gas mixes. During experimentation, animal breathing caused pressure fluctuations due to humidity and warmth of air in each expired breath, which were compared to the pressure of an identical reference chamber. Continuous monitoring of pressure differences between these two chambers by a differential pressure transducer (Validyne, Northridge, CA, United States), connected between the animal and reference chamber, allowed detection of breaths.

Oxygen consumption and CO_2_ production ($\dot{\text{V}}$_CO2_) rates were measured by analyzing the outflowing composition of gas by a Sable Systems FC-10 O_2_ analyzer and a Sable Systems CA-10 CO_2_ analyzer, respectively, and comparing outflowing gas concentrations to inflowing gas concentrations. The $\dot{\text{V}}$O_2_ was calculated from the product of the constant airflow through the chamber and the difference between the inflow and outflow in the fractional concentration of O_2_. The $\dot{\text{V}}$_CO2_ was calculated from the product of the constant airflow through the chamber and the difference between the outflow and inflow in the fractional concentration of CO_2_. All metabolic variables are reported at STPD.

Respiratory frequency (f_R_) was determined by counting $\dot{\text{V}}$_E_-induced pressure oscillations, whereas V_T_ was determined by integrating expiratory flow and then calculated using the method described by Drorbaugh and Fenn modified for open-flow plethysmography by Jacky (Drorbaugh and Fenn, 1955; Jacky, 1978). Pressure calibrations were performed prior to trials to determine V_T_ by injecting and withdrawing a known volume (0.2, 0.3, and 0.4 ml) into the experimental chamber at a rate similar to the respiration rate of the animal. Ventilation was calculated as the product of f_R_ and V_T_. All ventilatory measurements were selected when animals were resting and are reported at body temperature and pressure, saturated (BTPS).

### Air Convection Requirement, O_2_ Delivery and Lung O_2_ Extraction

The air convection requirement (ACR) for O_2_ (ACR_O2_; the quotient of $\dot{\text{V}}$_E_ and $\dot{\text{V}}$O_2_), the ACR for CO_2_ (ACR_CO2_; the quotient of $\dot{\text{V}}$_E_ and $\dot{\text{V}}$_CO2_), O_2_ delivery, and lung O_2_ extraction were calculated from ventilatory and metabolic measurements. Oxygen delivery to the lungs was calculated as ventilation multiplied by the fractional concentration of O_2_ in inspired air. The percent of O_2_ extracted from each breath was calculated by dividing $\dot{\text{V}}$O_2_ by $\dot{\text{V}}$_E_, multiplied by the fractional concentration of O_2_ in inspired air and multiplying that by 100.

### Experimental Design and Pharmacology

Control and CSH animals were transferred to the animal chamber under normoxic conditions and baseline recordings were obtained for 1 h. Next, animals were removed from the chamber and received ∼400 μL intraperitoneal injections of 0.9% NaCl saline alone (sham), dizocilpine (MK-801; 0.15 mg ⋅ kg^-1^), or cyanquixaline (6-cyano-7-nitroquinoxaline-2, 3-dione; CNQX; 5 mg ⋅ kg^-1^). Animals were then placed back in the animal chamber and $\dot{\text{V}}$_E_, $\dot{\text{V}}$_O2_, $\dot{\text{V}}$_CO2_, and body temperature were monitored for 1 h to assess the effect of the injection. Next, the inflowing gas composition was switched to 7% O_2_ (hypoxia) and these physiological variables were measured for 1 h. Following experimentation, animals were allowed to recover in normoxia and then returned to their colonies. CNQX and MK-801 were purchased from Sigma-Aldrich (St Louis, MO, United States).

### Data Collection and Statistical Analysis

All signals (body temperature, incurrent and excurrent O_2_ and CO_2_ concentrations, and the $\dot{\text{V}}$_E_ -induced pressure signal) were amplified, filtered, recorded and analyzed using PowerLab data acquisition hardware and LabChart software (AD Instruments Pty Ltd., Colorado Springs, CO, United States). From the recorded signals, and calculated dependent variables, we determined average: body temperature, $\dot{\text{V}}$_O2_, $\dot{\text{V}}$_CO2_, f_R_, V_T_, $\dot{\text{V}}$_E_, ACR_O2_, ACR_CO2_, O_2_ delivery and lung O_2_ extraction, for the last 10–15 min of each O_2_ exposure (21% O_2_ pre-injection, 21% O_2_ post-injection, and 7% O_2_ post-injection) to ensure animals reached a steady state. Inflowing gas concentrations were measured before and after each O_2_ exposure.

Statistical analyses were performed using R (R Core Team, 2017). We used linear mixed effects models (lme4 and lsmeans package; Bates et al., 2015; Lenth, 2016) to account for repeated sampling of the same individual with changes in O_2_ exposure, with individual treated as a random effect. When visual inspection of residuals, and q-q plots revealed deviations from the assumptions of linear mixed effects models (normality, homogeneity of variances, linearity, and independence), we log transformed the dependent variable. We entered acclimation group (control or CSH), drug treatment (sham or drug), level of inspired O_2_ (21% O_2_ or 7% O_2_), and body mass as fixed effects in our initial models. We tested all 2- and 3-way interactions of acclimation group, drug treatment, and level of inspired O_2_. We did not remove any terms from our models given the importance of all independent variables and interactions to our research objectives. When interaction terms were significant the data were separated and analyzed independently using a one-way ANOVA, followed by a Tukey-Holm *post hoc* analysis to determine differences between acclimation group, drug treatment, level of inspired O_2_, and to correct for multiple pairwise comparisons. All results are presented as mean ± SD, with statistical significance set as *p* < 0.05. Results from statistical tests are included in the attached supplemental table (Supplementary Table S1).

## Results

### Naked Mole Rats Exhibit a Relative Hypoxic Ventilatory Response to Acute Hypoxia Masked by a Robust Hypoxic Metabolic Response

**FIGURE 1:**
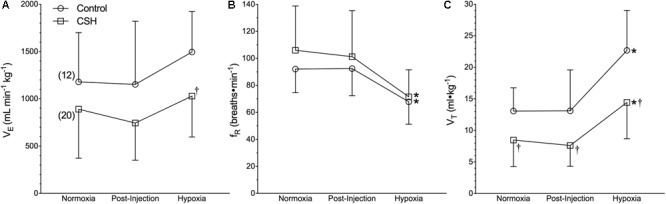
Ventilatory responses to acute hypoxia of naked mole rats acclimated to normoxia or chronic sustained hypoxia (CSH). **(A–C)** Summaries of total minute ventilation ($\dot{\text{V}}$_E_; **A**); breathing frequency (f_R_; **B**), and tidal volume (V_T_; **C**) from naked mole rats exposed to 21% O_2_, before and after sham intraperitoneal saline injections, and subsequent exposure to acute hypoxia (7% O_2_). Data are presented as mean ± SD. Numbers in parenthesis indicate *n* values. ^∗^Indicate significant difference in acute hypoxia from normoxic controls. ^†^Indicate significant difference between sham-treated animals acclimated in chronic hypoxia (CSH) vs. normoxia; *P* < 0.05.

**FIGURE 2:**
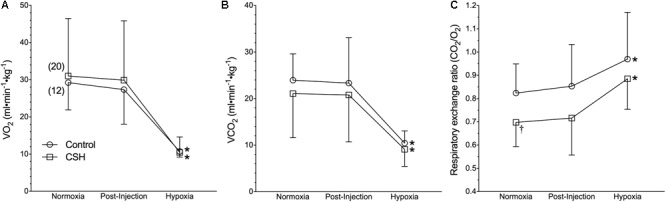
Naked mole rats exhibit a robust metabolic response to acute hypoxia that is not different between animals acclimated to normoxia or chronic sustained hypoxia (CSH). **(A–C)** Summaries of O_2_ consumption rate ($\dot{\text{V}}$_O2_; **A**); CO_2_ production rate ($\dot{\text{V}}$_CO2_; **B**), and the respiratory exchange ratio (RER; **C**) from naked mole rats exposed to 21% O_2_, before and after sham intraperitoneal saline injections, and subsequent exposure to acute hypoxia (7% O_2_). Data are presented as mean ± SD. Numbers in parenthesis indicate *n* values. ^∗^Indicate significant difference in acute hypoxia from normoxic controls. *P <* 0.05.

**FIGURE 3:**
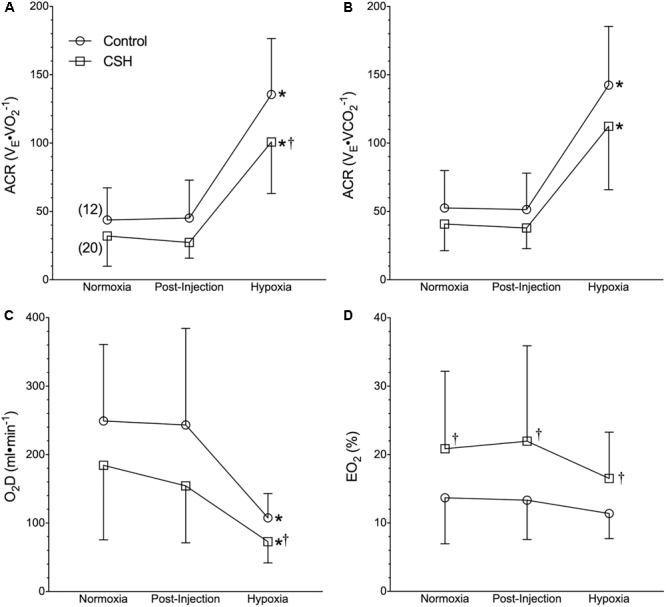
Naked mole rats exhibit a relative hypoxic ventilatory response. **(A–D)** Summaries of the air convection requirement to O_2_ (ACR_O2_; **A**); the air convection requirement to CO_2_ (ACR_CO2_; **B**), the rate of O_2_ delivery (DO_2_; **C**), and the O_2_ extraction percentage (E_O2_%; **D**) from naked mole rats exposed to 21% O_2_, before and after sham intraperitoneal saline injections, and subsequent exposure to acute hypoxia (7% O_2_). Data are presented as mean ± SD. Numbers in parenthesis indicate *n* values. ^∗^Indicate significant difference in acute hypoxia from normoxic controls. ^†^Indicate significant difference between sham-treated animals acclimated in chronic hypoxia (CSH) vs. normoxia; *P <* 0.05.

Our first objective was to revaluate the acute hypoxic ventilatory response of naked mole rats exposed to acute hypoxia using body temperature measurements obtained non-invasively from awake and freely behaving animals. Sham injections had no effect on any ventilatory or metabolic variable examined from control animals (Figure 1–3, open circles; *n* = 12; note: all statistical test results are included in Supplementary Table S1). Acute hypoxia did not elicit a significant change in $\dot{\text{V}}$_E_ (Figure 1A), although the components of $\dot{\text{V}}$_E_ (f_R_ and V_T_) changed significantly, but in opposite directions from one another. Specifically, and relative to pre-injection normoxic controls, f_R_ was 26% lower in acute hypoxia (Figure 1B), whereas V_T_ was 73% higher (Figure 1C).

On the other hand, acute hypoxia elicited a robust hypoxic metabolic response. Relative to values obtained in normoxic control animals, $\dot{\text{V}}$*_O2_* and $\dot{\text{V}}$_CO2_ were reduced by 62 and 57%, respectively in acute hypoxia (Figure 2A,B). Control naked mole rats exhibited a consistent shift in their respiratory exchange ratio (RER) from 0.82 in normoxia to 0.97 in acute hypoxia (Figure 2C), indicating the occurrence of a metabolic fuel switch toward a greater reliance on carbohydrate metabolism.

Although control naked mole rats did not exhibit a significant change in absolute $\dot{\text{V}}$_E_ when breathing acute hypoxia, as a result of their robust metabolic rate depression, their ACR increased ∼3-fold (Figure 3A,B), indicative of a relative hyperventilation. Despite this, O_2_ delivery was significantly reduced by 56% during acute hypoxia (Figure 3C), and lung O_2_ extraction from the inspired air was unchanged (Figure 3D).

### Naked Mole Rats Exhibit Ventilatory but Not Metabolic Plasticity Following Chronic Sustained Hypoxic Exposure

Next, we examined the effect of 8*–*10 days of CSH on naked mole rat $\dot{\text{V}}$_E_ and metabolism. Ventilatory acclimatization, when it occurs, remains transiently for days when animals are returned to breathing normoxic gas mixtures. In naked mole rats, we found a significant effect of chronic acclimation in hypoxia on $\dot{\text{V}}$_E_; however, $\dot{\text{V}}$_E_ was not significantly different in CSH animals breathing normoxic gas mixtures 1 h post-CSH compared to control animals (Figure 1A). Breathing frequency was not different between control and CSH animals (Figure 1B); however, V_T_ was ∼35% lower in CSH animals relative to control animals when breathing normoxic gas (Figure 1C), indicating that some degree of ventilatory remodeling occurred during CSH in this species, albeit in the opposite direction of that which occurs in all other mammalian adults.

Interestingly, when breathing acute hypoxic gas, CSH animals exhibited a similar (29%) decrease in f_R_ and increase (47%) in V_T_, compared to control animals (Figure 1B,C). In this case, however, the net effect was that absolute $\dot{\text{V}}$_E_ in CSH animals breathing acute hypoxia was significantly higher than in normoxia. Conversely, acclimation to CSH had no significant effect on $\dot{\text{V}}$_O2_ or $\dot{\text{V}}$_CO2_ in normoxia or on the hypoxic metabolic response, which were similar in magnitude to those of control animals (Figure 2A,B). However, due to small but insignificant changes in $\dot{\text{V}}$_O2_ and $\dot{\text{V}}$_CO2_, the RER of CSH-acclimated animals breathing normoxia was significantly lower than that of control animals, magnifying the hypoxic increase in the RER (Figure 2C).

Due to the smaller increase in V_T_ (and thus $\dot{\text{V}}$_E_) in CSH animals, the ACR of CSH-acclimated naked mole rats was also slightly smaller than that of control animals in acute hypoxia (Figure 3A,B). However, the net change in the ACR with hypoxia was similar between these groups (Figure 3A,B). The differences in ventilation between treatment groups resulted in a reduction in the rate of O_2_ delivery in CSH animals that was significant during acute hypoxic exposure but not in normoxia (Figure 3C). Conversely, the lung O_2_ extraction from the air was ∼1.5 to 2-fold higher in CSH animals in both normoxia and acute hypoxia (Figure 3D).

### Glutamate Receptors Contribute to the Regulation of Ventilation and Metabolism in Normoxia but Not Hypoxia

**FIGURE 4:**
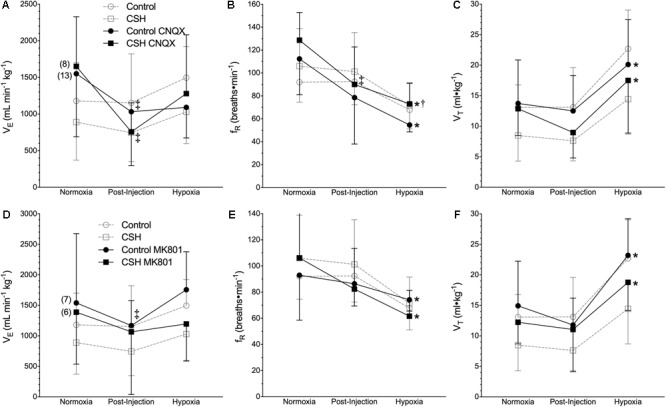
Inhibition of glutamatergic receptors modifies ventilation in normoxia but not in acute hypoxia in naked mole rats. **(A–F)** Summaries of total minute ventilation ($\dot{\text{V}}$_E_; **A,D**); breathing frequency (f_R_; **B,E**), tidal volume (V_T_; **C,F**) from naked mole rats exposed to 21% O_2_, before and after intraperitoneal injections of the AMPAR antagonist 6-cyano-7-nitroquinoxaline-2,3-dione; (CNQX; 5 mg ⋅ kg^-1^ **A–C**) or the NMDAR antagonist dizocilpine (MK-801; 0.15 mg ⋅ kg^-1^; **D–F**), and subsequent exposure to acute hypoxia (7% O_2_). Data are presented as mean ± SD. Numbers in parenthesis indicate *n* values. ^∗^Indicate significant difference in acute hypoxia from normoxic controls. ^†^Indicate significant difference between drug-treated animals acclimated in chronic hypoxia (CSH) vs. normoxia. ^‡^Indicate significant difference between pre- and post-injection groups; *P <* 0.05.

We next explored the potential role for excitatory glutamate receptor signaling in mediating the acute hypoxic ventilatory response and ventilatory acclimatization to hypoxia. Unlike sham injections (Figure 1–3), blockade of AMPARs with CNQX reduced $\dot{\text{V}}$_E_ immediately following drug injection in normoxia in both groups [Figure 4A; *n* = 13 for control + CNQX (closed circles) and 8 for CSH + CNQX (closed squares); note, sham injections are shown in gray for reference]. These changes were due to a combination of mostly non-significant reductions in both f_R_ and V_T_ following CNQX injection, in both control and CSH groups (Figure 4B,C); only the reduction in f_R_ in control animals was significant. NMDAR blockade with MK801 also significantly reduced $\dot{\text{V}}$_E_ but only in control animals [Figure 4D–F; *n* = 7 for control + MK801 (closed circles) and 6 for CSH + MK801 (closed squares)].

When animals in each group were then exposed to acute hypoxia, we did not observe any significant effects of either drug treatment (CNQX or MK801) on any ventilatory variable; the hypoxic changes in f_R_ and V_T_ in both groups were similar in magnitude to those of the control animals. However, while V_T_ was still reduced in the CSH group relative to the control group in both CNQX- and MK801-treated animals in both normoxia and acute hypoxia, the difference was no longer significant.

**FIGURE 5:**
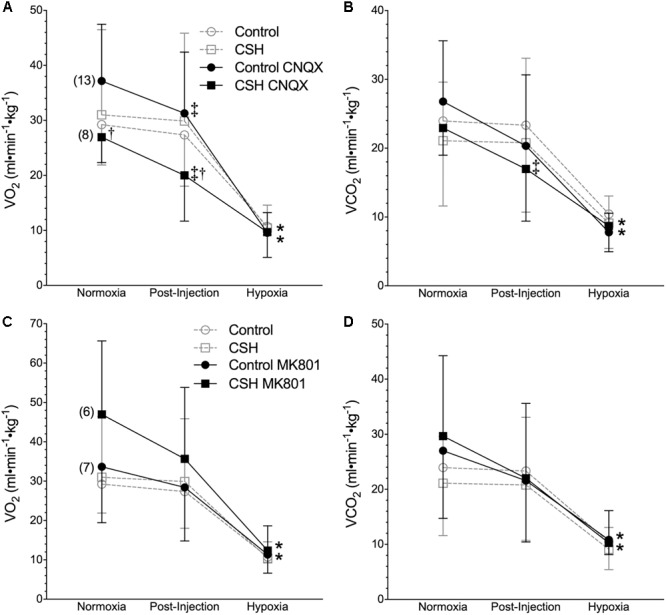
Inhibition of glutamatergic receptors modifies metabolic rate in normoxia but does not affect metabolic responses to acute hypoxia in naked mole rats. **(A–D)** Summaries of O_2_ consumption rate ($\dot{\text{V}}$_O2_; **A,C**); and CO_2_ production rate ($\dot{\text{V}}$_CO2_; **B,D**) from naked mole rats exposed to 21% O_2_, before and after intraperitoneal injections of the AMPAR antagonist 6-cyano-7-nitroquinoxaline-2,3-dione; (CNQX; 5 mg⋅kg^-1^; **A,B**) or the NMDAR antagonist dizocilpine (MK-801; 0.15 mg⋅kg^-1^; **C,D**), and subsequent exposure to acute hypoxia (7% O_2_). Data are presented as mean ± SD. Numbers in parenthesis indicate *n* values. ^∗^Indicate significant difference in acute hypoxia from normoxic controls. ^†^Indicate significant difference between drug-treated animals acclimated in chronic hypoxia (CSH) vs. normoxia. ^‡^Indicate significant difference between pre- and post-injection groups; *P <* 0.05.

**FIGURE 6:**
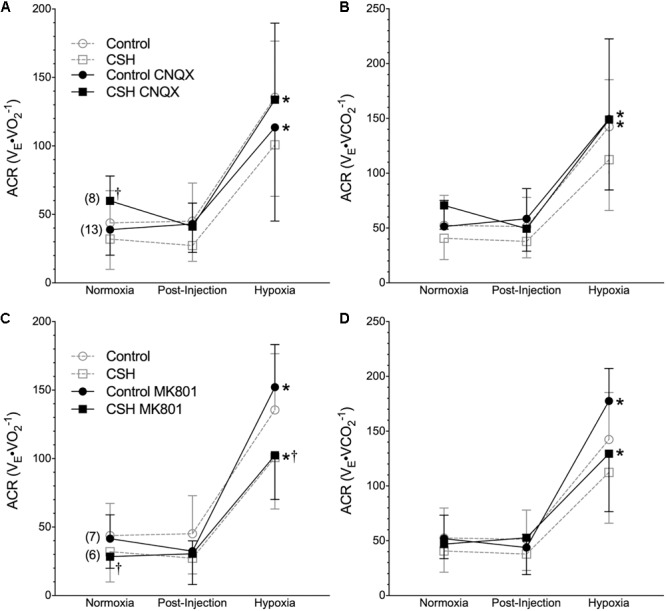
Inhibition of glutamatergic receptors does not enhance the hypoxic ventilatory response of naked mole rats breathing acute hypoxia. **(A–D)** Summaries of the air convection requirement to O_2_ (ACR_O2_; **A,C**); the air convection requirement to CO_2_ (ACR_CO2_; **B,D**), from naked mole rats exposed to 21% O_2_, before and after intraperitoneal injections of the AMPAR antagonist 6-cyano-7-nitroquinoxaline-2,3-dione; (CNQX; 5 mg⋅kg^-1^; **A,B**) or the NMDAR antagonist dizocilpine (MK-801; 0.15 mg⋅kg^-1^, **C,D**), and subsequent exposure to acute hypoxia (7% O_2_). Data are presented as mean ± SD. Numbers in parenthesis indicate *n* values. ^∗^Indicate significant difference in acute hypoxia from normoxic controls. ^†^Indicate significant difference between drug-treated animals acclimated in chronic hypoxia (CSH) vs. normoxia; *P <* 0.05.

Metabolic rate (i.e., $\dot{\text{V}}$_O2_ and $\dot{\text{V}}$_CO2_) was also reduced following injection of either CNQX or MK801 in both control and CSH groups during normoxia, although this effect was only significant in the CNQX-treated animals (Figure 5A–D). However, neither CNQX nor MK801 treatment affected the acute hypoxic metabolic response in either group. Finally, glutamate receptor inhibition had little effect on the ACR_O2_ and ACR_CO2_ during acute hypoxia in either control or CSH group (Figure 6).

## Discussion

In the present study we set out to re-evaluate the acute hypoxic ventilatory response and the occurrence of ventilatory acclimatization to chronic hypoxia in naked mole rats using more accurate body temperature measurements than in previous studies. We also investigated the potential role of excitatory glutamatergic signaling in the hypoxic ventilatory response with and without acclimation to CSH. Our study yielded four important findings. First, with the incorporation of more accurate body temperature measurements in our calculations of V_T_, we report that naked mole rats do not reduce $\dot{\text{V}}$_E_ when acutely breathing hypoxic gas following acclimation to CSH. They do not increase $\dot{\text{V}}$_E_ either, but, breathing patterns are altered by acute hypoxia such that f_R_ decreases and V_T_ increases in an offsetting fashion, and $\dot{\text{V}}$_E_ remains unchanged. Second, naked mole rats exhibit a robust hypoxic metabolic response, and as a result, they hyperventilate (i.e., express a relative hypoxic ventilatory response, as indicated by an increase in their ACR). Third, naked mole rats exhibit an atypical form of ventilatory acclimatization to hypoxia in which levels of V_T_ and $\dot{\text{V}}$_E_ *decrease* following CSH, while metabolism is not affected by acclimation to CSH. Finally, glutamatergic inhibition reduces $\dot{\text{V}}$_E_ through a decrease in f_R_, and also reduces metabolic rate in normoxia in both control and CSH groups. However, this intervention does not influence ventilatory or metabolic responses to acute hypoxia in either the control or CSH group. To our knowledge, a lack of a role for glutamatergic signaling in mediating the hypoxic ventilatory response is unique among adult mammals.

### Effects of Hypoxic Changes in Body Temperature Measurements on Tidal Volume Calculations

Our earlier studies and those from other laboratories reported that naked mole rats do not exhibit a significant decrease in body temperature in response to acute hypoxic or anoxic exposure (Nathaniel et al., 2012; Pamenter et al., 2015a; Chung et al., 2016; Park et al., 2017). It now appears that the body temperature measurements in these studies were confounded, likely by heat transfer during the experimental handling of awake animals to measure rectal temperature (in our own studies), or the use of anesthetics (in studies from other laboratories), which reduce brown adipose tissue thermogenesis (Tatsumi et al., 2004; Zhang et al., 2013). We recently reported that awake, freely behaving naked mole rats rapidly reduce their body temperature during acute hypoxia (Ilacqua et al., 2017; Kirby et al., 2018), and reconfirm this finding here. In the present study, at an ambient temperature of 29.0°C, body temperature decreased from 32.0 ± 0.2 to 30.5 ± 0.2°C in acute hypoxia. Relative to most other adult mammals, naked mole rats are poor thermoregulators and have a resting body temperature that is typically 1–2°C above ambient temperature when held near their thermoneutral zone (31–34°C) (Buffenstein and Yahav, 1991; Yahav and Buffenstein, 1991). While this does not leave much room for body temperature to fall, the decrease measured here is important. Accurate measurements of body temperature are critical to the calculation of V_T_ and therefore $\dot{\text{V}}$_E_ when using barometric plethysmography (Mortola and Frappell, 1998, 2013). When we use the body temperature values from the present study in our calculations of V_T_, we find that relative to pre-injection normoxic controls, f_R_ is 26% lower in acute hypoxia (Figure 1B), whereas V_T_ is 73% higher (Figure 1C), while acute hypoxia does not elicit a significant change in $\dot{\text{V}}$_E_ (Figure 1A). If we do not correct for the small fall in body temperature, we see a smaller increase in V_T_, and the same fall in $\dot{\text{V}}$_E_ reported in earlier studies (data not shown).

### Acute Hypoxia Alters Breathing Pattern but Not Total Ventilation While Chronic Sustained Hypoxia Reduces Total Ventilation

A central finding of our study is that CSH significantly reduces V_T_ and $\dot{\text{V}}$_E_ in naked mole rats. This was surprising given that most adult mammals exhibit an *increase* in $\dot{\text{V}}$_E_ after CSH (Pamenter and Powell, 2016), not a *decrease*. In all other adult mammals, the manifestation of ventilatory acclimatization to hypoxia is driven by a combination of increased sensitivity to inspired O_2_ at the carotid bodies and to afferent excitatory glutamatergic signals at the synaptic connections with the carotid sinus nerve within the NTS (i.e., CNS gain; Kumar and Prabhakar, 2012; Pamenter and Powell, 2016). While the reverse phenotype seen in adult naked mole rats may represent upregulation of inhibitory neurotransmission at the carotid body or the NTS, or both, it may also reflect the observed lack of involvement of excitatory glutamatergic signaling in the hypoxic ventilatory response (see below). Regardless, in both control and CSH animals, acute hypoxia alters breathing pattern (a reduction in f_R_ and an increase in V_T_) but not $\dot{\text{V}}$_E_.

### The Relative Hypoxic Ventilatory Response Is Driven Entirely by a Robust Hypoxic Metabolic Response

We observe that control naked mole rats undergo a robust decrease in their $\dot{\text{V}}$_O2_ during acute hypoxia of ∼65% relative to normoxic values. This change is consistent with measurements in similarly treated naked mole rats [65% (Chung et al., 2016) and 70% (Pamenter et al., 2015a)] Robust metabolic rate depression during acute hypoxia is a hallmark response of neonatal rodents (Bonora et al., 1984; Mortola et al., 1989; Mortola, 2004). In neonatal rodents, the hypoxia-mediated fall in metabolic rate is primarily due to a switching off of non-shivering thermogenesis (Mortola, 1999). Naked mole rats are poor thermoregulators but they do possess brown adipose tissue (Daly et al., 1997), and in light of the neotenic changes in breathing pattern and metabolism we see during acute hypoxia in this study, it is conceivable that shutting down brown adipose tissue-mediated non-shivering thermogenesis may play a role in the hypoxic decreases in both $\dot{\text{V}}$_O2_ and body temperature.

Naked mole rats also exhibit a fuel switch toward increased reliance on carbohydrates with acute hypoxia. This response is similar to that observed from other hypoxia-adapted rodents, including murine species that live at high altitudes (Schippers et al., 2012). The ATP yield per mole of O_2_ catabolized is 15–30% higher when derived from carbohydrates than from lipids (due to the higher energetic costs of breaking high energy bonds in lipids) and thus a greater reliance on carbohydrate fuels would increase energetic efficiency during acute hypoxia. This strategy would be particularly useful in naked mole rats because they likely experience hypoxia transiently during intense exercise and when resting (e.g., when digging tunnels or sleeping, respectively) and thus would have opportunities to replenish carbohydrate stores while in more normoxic regions of their burrows. It is in these normoxic regions that they rely more heavily on lipid energy stores (as indicated from our normoxic RER calculations). In CSH animals we observe a greater reliance on lipids, suggesting that some form of metabolic remodeling occurs during CSH. Endogenous carbohydrate stores are unlikely to be sufficient to sustain metabolic needs during prolonged hypoxia, likely leading to the reversal in fuel use compared to what is seen in acute hypoxia.

### Glutamatergic Receptors Are Not Involved in the Naked Mole Rat’s Hypoxic Ventilatory Response

We report that glutamatergic receptor inhibition reduces ventilation and metabolic rate in normoxia but does not impact the acute hypoxic ventilatory response or ventilatory acclimatization to hypoxia. These findings were surprising because excitatory glutamatergic signaling is the primary neurotransmission pathway that underlies the acute hypoxic ventilatory response and ventilatory acclimatization to hypoxia in most other adult mammalian species (Pamenter and Powell, 2016). Conversely, in neonatal rodents a biphasic hypoxic ventilatory response is observed that is primarily mediated by inhibitory adenosinergic signaling (Elnazir et al., 1996; Johansson et al., 2001). We have previously demonstrated that the acute hypoxic ventilatory response of naked mole rats is also mediated by inhibitory adenosinergic signaling (Pamenter et al., 2015a). It is important to note that naked mole rats do express NMDARs within their CNS (Peterson et al., 2012); however, our current study indicates that these receptors are not involved in the hypoxic ventilatory response. This finding is consistent with the lack of a significant increase in $\dot{\text{V}}$_E_ under any hypoxic condition in naked mole rats, which indicates a potential deficit of function in excitatory signaling mechanisms related to the control of breathing.

### Study Limitations

The fact that we observe effects of CNQX and MK801 on breathing and metabolic rate in normoxia suggests that these drugs and the concentrations we employed in our study are efficacious in this species. However, glutamatergic receptor antagonists were injected intraperitoneally and thus their specificity of action must be interpreted with caution. Unfortunately, the anatomy of naked mole rats, and specifically the location of large muscle masses on the top of the cranium, which are critical for eating, digging, and other social functions within the colony, makes stereotaxic implantation of permanent cannulas that target the respiratory brainstem detrimental to the health and sociability of this species. However, intraperitoneal injections of these same pharmacological agents have been used previously in numerous studies in mice and rats to evaluate a wide variety of physiological and behavioral responses (for example: Velisek et al., 1995; Mead and Stephens, 1999; Berrino et al., 2003; Murschall and Hauber, 2005; Reid and Powell, 2005; McGuire et al., 2008; Jeon et al., 2017; Xiang et al., 2018). This large body of literature in which these drugs impacted physiological function following intraperitoneal injection (as in our study) indicates that these agents successfully cross the blood brain barrier to interact with AMPARs and NMDARs, which are not found outside of the CNS. Importantly, a handful of studies have successfully utilized intraperitoneal injections of MK801 to investigate the role of NMDARs in the hypoxic ventilatory responses of rats (Reid and Powell, 2005; McGuire et al., 2008), and these results have been subsequently supported by similar findings using microinjection techniques targeted specifically to respiratory brainstem regions (Pamenter et al., 2014a). It is also important to note when either AMPARs or NMDARs were antagonized in isolation, the other glutamatergic receptors that was not targeted may have compensated to some degree, although the impact of this is difficult to predict.

## Conclusion

In the present study we demonstrate that naked mole rats do not alter $\dot{\text{V}}$_E_ in response to acute hypoxia but nonetheless mount a relative hypoxic ventilatory response, as indicated by an increase in their ACR, mediated by a robust depression of $\dot{\text{V}}$_O2_. There is debate regarding the degree to which naked mole rats experience hypoxia in their day to day lives, with a recent study indicating that their burrows are not particularly hypoxic (Holtze et al., 2018). However, measurements in this study were limited to tunnel regions near recent burrow openings and naked mole rats likely do experience significant periods of hypoxia at their metabolic extremes: both within their crowded and poorly ventilated nest chambers while sleeping and resting, and when working at their aerobic limit to dig and chew through densely packed soils. We speculate that the lack of a net ventilatory response to acute hypoxia represents an adaptation to regular exposures to intermittent and variable periods of hypoxia, and effectively results in a smoothed ventilatory phenotype in variable burrow O_2_ conditions. The apparent lack of a role for glutamatergic signaling in the naked mole rat ventilatory response to acute or chronic hypoxia is consistent with this speculation because repeated or prolonged activation of glutamatergic pathways is typically associated with synaptic plasticity and sustained changes in the sensitivity of an organism to environmental stimuli (i.e., ventilatory acclimatization to hypoxia), which would be undesirable and energetically expensive for an organism that frequently experiences variable levels of hypoxia while engaging in highly divergent activity states.

## Author Contributions

MP, AS, and WM conceived of and designed the study. MP, WM, and YD wrote the manuscript. SG, AS, DC, YD, MP, and LB performed the experiments. YD analyzed the data. All authors gave final approval of the published version and agree to be accountable for all content therein.

## Conflict of Interest Statement

The authors declare that the research was conducted in the absence of any commercial or financial relationships that could be construed as a potential conflict of interest.

## Supplementary Material

TABLE S1 Linear mixed effects models for all dependent variables presented in the main body of the text used to test for effects of acclimation group [control or chronic sustained hypoxia (CSH)], drug treatment [sham or drug (CNQX and MK-801)], and level of inspired O_2_ (21% O_2_ or 7% O_2_). Statistical significance is set as *p* < 0.05 and indicated by the dark gray boxes.

## Abbreviations

ACR: air convection requirement
AMPAR: α-amino-3-hydroxy-5-methyl-4-isoxazolepropionic acid receptor
CNQX: cyanquixaline (6-cyano-7-nitroquinoxaline-2,3-dione)
CNS: central nervous system
CSH: chronic sustained hypoxia
f_R_: breathing frequency
MK-801: dizocilpine
NMDAR: N-methyl-D-aspartate receptor
NTS: nucleus of the solitary tract (*nucleus tractus solitarius*)
Pa_O2_: partial pressure of arterial oxygen
$\dot{\text{V}}$_E_: minute ventilation
$\dot{\text{V}}$_CO2_: rate of carbon dioxide production
$\dot{\text{V}}$_O2_: oxygen consumption rate
V_T_: tidal volume
